# MICA/B expression is inhibited by unfolded protein response and associated with poor prognosis in human hepatocellular carcinoma

**DOI:** 10.1186/s13046-014-0076-7

**Published:** 2014-09-18

**Authors:** Liang Fang, Jiuyu Gong, Ying Wang, Rongrong Liu, Zengshan Li, Zhe Wang, Yun Zhang, Chunmei Zhang, Chaojun Song, Angang Yang, Jenny P -Y Ting, Boquan Jin, Lihua Chen

**Affiliations:** Department of Immunology, the Fourth Military Medical University, Xi’an, 710032 Shaanxi China; Hospital of Hubei Armed Police Corps, Wuhan, Hubei China; Department of Pathology, the Fourth Military Medical University, Xi’an, Shaanxi China; Department of Microbiology and Immunology, University of North Carolina at Chapel Hill, Chapel Hill, NC 27599 USA

**Keywords:** MICA/B, Hepatocellular carcinoma, Natural killer cell, Unfolded protein response, Innate immunity

## Abstract

**Background:**

MICA/B are major ligands for NK cell activating receptor NKG2D and previous studies showed that the serum level of soluble MICA (sMICA) is an independent prognostic factor for advanced human hepatocellular carcinoma. However, the correlation between cellular MICA/B expression pattern and human hepatocellular carcinoma progression has not been well explored. The unfolded protein response is one of the main causes of resistance to chemotherapy and radiotherapy in tumor cells. However, whether the UPR in HCC could regulate the expression levels of MICA/B and affect the sensitivity of HCC cells to NK cell cytolysis has not been established yet.

**Methods:**

MICA/B expression pattern was evaluated by immunohistochemistry and Kaplan-Meier survival analysis was done to explore the relationship between MICA/B expression level and patient survival. The protein and mRNA expression levels of MICA/B in SMMC7721 and HepG2 cells treated by tunicamycin were evaluated by flow cytometry, Western Blot and RT-PCR. The cytotoxicity analysis was performed with the CytoTox 96 Non-Radioactive LDH Cytotoxicity Assay.

**Results:**

MICA/B was highly expressed in human hepatocellular carcinoma and the expression level was significantly and negatively associated with tumor-node metastasis (TNM) stages. *Patients with low level of MICA/B expression showed a trend of shorter survival time*. The unfolded protein response (UPR) downregulated the expression of MICA/B. This decreased protein expression occurred via post-transcriptional regulation and was associated with proteasomal degradation. Moreover, decreased expression level of MICA/B led to the attenuated sensitivity of human HCC to NK cell cytotoxicity.

**Conclusion:**

These new findings *of the connection of MICA/B, UPR and NK cells may represent a new concrete theory of NK cell regulation in HCC,* and suggest that targeting this novel NK cell-associated immune evasion pathway may be meaningful in treating patients with HCC.

**Electronic supplementary material:**

The online version of this article (doi:10.1186/s13046-014-0076-7) contains supplementary material, which is available to authorized users.

## Introduction

Hepatocellular carcinoma (HCC) is the third most common of cancer-related deaths worldwide and accounts for approximately 70%-80% of all primary liver cancer cases [1]. Currently, removal of the malignant tissue by surgical resection is the main line of treatment. However, recurrence is quite common in patients who have had a resection, and the results of current therapy for advanced disease are poor with 30%-40% 5 years survivals [2]. Prevention of HCC progression and its recurrence is one of the most challenging aspects for efficient cancer immunotherapy of HCC.

NK cells mediate direct cytotoxic activity against tumor cells and provide the early host defense against the neoplastic cells. Although there are enriched NK cells in hepatoma tissues and NK cells within a healthy liver exhibit a higher level of cytotoxicity against tumor cells and express higher levels of cytotoxicity mediators when compared with peripheral NK cells [3,4], hepatoma cells still could escape from NK cell-mediated killing and survive. We conjectured that there are likely undefined molecular and cellular mechanisms by which hepatoma cells escape from NK cell recognition and killing.

The interaction between NK cell activating receptors and their ligands are known to modulate immune surveillance and affect the tumor progression. Major histocompatibility complex class I-related chain A and B (MICA/B) are two stress-inducible ligands that bind to the immunoreceptor, NKG2D and play an important role in mediating cytotoxicity of NK cells [5]. Although expression of MICA/B in normal tissues is restricted mainly to the thymus and gastrointestinal epithelium, MICA/B were detected on several carcinoma cells such as liver, lung, breast, ovary, prostate and colon cancer [6,7]. The cell surface expression of MICA/B is regulated by heat shock, viral or bacterial infections or pharmacological agents which are known to affect the antitumor immunity [5,8,9]. This raises the possibility that MICA/B may be specific targets on tumor cells for NK-mediated cytolytic activity.

*The unfolded protein response (UPR) is a consequence of endoplasmic reticulum (ER) stress which is triggered by accumulation of incorrect folding and improper glycosylation of newly synthesized proteins in ER. The UPR induced by the stressors contributes to the survival, growth, progression and chemo-resistance of cancer* [10]*. It has also been demonstrated that UPR may be a protective mechanism for tumor.* HCC progression is often accompanied by hypoxia, decreased glucose supply and increased UPR in the tumor microenvironment [11-13]. However, whether the UPR in HCC could regulate the expression levels of the ligands for NK cell activating receptors and affect the sensitivity to NK cell cytolysis has not been established yet.

In this study, we systematically investigated the protein expression of MICA/B in 5 normal liver tissues and 96 hepatocellular carcinoma tissues. We also studied the value of MICA/B for prognosis in HCC patients and showed that the expression levels of MICA/B on hepatoma cells were significantly down-regulated during UPR, which consequently led to more resistance of hepatoma cells to NK cell cytotoxicity.

## Methods

### Patients and follow-up

The study was approved by the Ethics Committee at FMMU, and informed consent was obtained from each patient. A total of 96 histologically confirmed HCC patients undergoing radical resection at 2008 were enrolled from Xijing hospital of the Fourth Military Medical University. None of these patients had received any anti-cancer therapy before surgery. The TNM stage was determined according to the 7th edition UICC/AJCC staging system. Patients were followed from the time of hospital admission and first treatment until August, 2012. Overall survival (OS), defined as the time from operation to death or last follow-up, was used as a measure of prognosis.

### Immunohistochemical (IHC) staining and evaluation

Immunohistochemical detection of MICA/B was done as described previously [14,15]. Briefly, paraffin sections of human hepatoma tissues were dewaxed, hydrated and incubated in peroxidase inhibitor for 30 min to remove endogenous peroxidase. The samples were then dipped into rabbit anti-human MICA/B polyclonal antibody (Abgent, diluted to 1:100) at 4°C overnight after blocking with diluted goat serum (Sigma, MO, USA). Rabbit IgG prior to immunization and antigen-absorbent immune serum were applied as negative controls. After three washes in PBS, the slices were dipped into HRP-conjugated goat anti-mouse IgG/anti-rabbit IgG (Abcam, MA, UK) for 30 min at room temperature. The antibody complexes were then visualized by incubation with diaminobenzidine chromogen. The sections were counterstained with Mayer’s hematoxylin for 2 min, dehydrated through a graded ethanol series, cleared in dimethyl benzene, mounted and examined using light microscopy (Olympus, Tokyo, Japan).

All slices were evaluated by two pathologists without knowledge of the clinical outcome. The percentage of immune-reactive cells and the staining intensities were evaluated in each sample. The percentage of immune-reactive cells was graded on a scale of 0 to 4, where no staining was scored as 0; 1–10% of cells stained was scored as 1; 11–50% was scored as 2; 51–80% was scored as 3; and 81–100% was scored as 4. The staining intensities were graded from 0 to 3, where 0 was defined as negative; 1 as weak; 2 as moderate; and 3 as strong. The total score was calculated as the product of intensity and percentage scores, ranging from 0 to 12. The expression level was divided as low or high by the median total score.

### Cell culture

NK cells used in functional experiments were isolated from healthy donors and resuspended in complete medium (RPMI 1640 containing 100 μg/ml L-glutamine, 10% heat-inactivated FBS, 100 U/ml penicillin G, and 100 μg/ml streptomycin) supplemented with 1000 IU/ml IL-2 and incubated overnight at 37°C before use. SMMC7721 cells were maintained in RPMI 1640 with 10% FBS and penicillin/streptomycin, and HepG2 cells were maintained in DMEM with 10% FBS and penicillin/streptomycin.

### Flow cytometry, western blot and cytotoxicity analyses

SMMC7721 and HepG2 cells were stained with anti-MICA/B (clone 6D4, Biolegend, CA, USA) for 30 min and fluorescein isothiocyanate (FITC)-labeled goat anti-mouse IgG1κ mAb (BD Biosciences, NJ, USA) for 20 min on ice. Mouse IgG1κ isotype control (BD Biosciences, NJ, USA) was used as a negative control. A minimum of 20,000 gated events/sample was collected on a flow cytometer (FACSCalibur, Elite ESP, FL, USA) and analyzed using CellQuest software. SMMC7721 and HepG2 cells from different treatment groups were lysed in lysis buffer (Bio-Rad, CA, USA), and the western blot analysis was performed as described previously [10]. The cytotoxicity analysis was performed with the CytoTox 96 Non-Radioactive LDH Cytotoxicity Assay (Promega, WI, USA), according to the protocol provided. To analyze the involvement of MICA/B in cytolytic activity of NK cells, anti-MICA/B mAb (6D4) or isotype-matched control Ab was added during the cytolytic assay.

### Statistical analyses

Each experiment was performed independently at least three times and one representative experiment is presented. All statistical analyses were performed using the SPSS 13.0 statistical software package. Average values are reported as the mean ± S.D. The significance of differences was analyzed statistically by the compared *t* test with Welch’s correction, or Mann-Whitney *U* test. The correlation between MICA/B level and GRP78 level was evaluated by correlation, and Pearson correlation coefficients were calculated to estimate the correlations. A Kaplan-Meier survival function was calculated and compared with a log-rank test to assess the differences of OS. All statistical tests were 2-sided, and P-values <0.05 were considered significant.

## Results

### Clinical significance of MICA/B expression profile in HCC tissue

**Figure 1:**
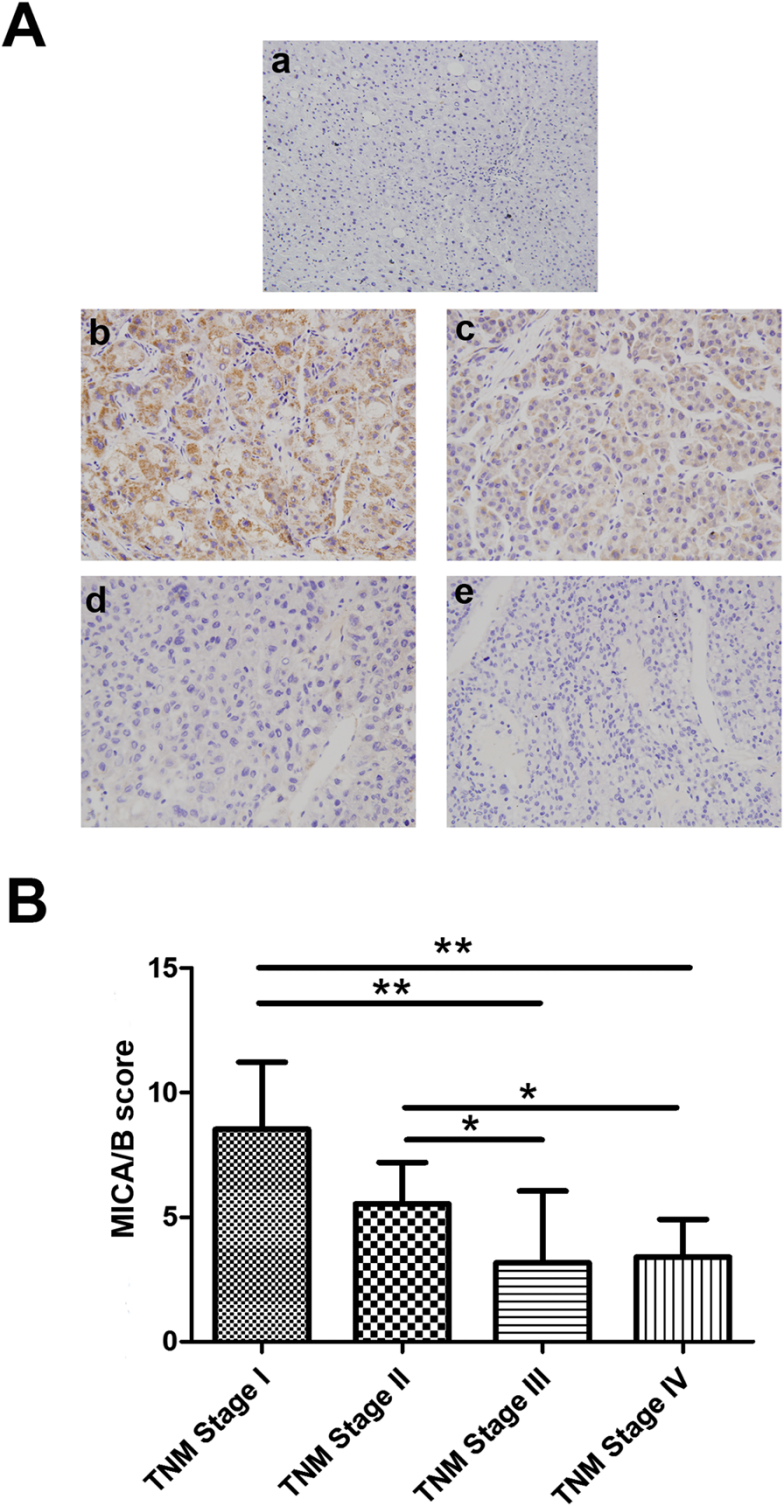
**Immunohistochemical staining of MICA/B in HCC patients with different clinical stages. (A)** Representative immune-histochemical staining of the expression of MICA/B in HCC tissues. a, normal liver tissue; b, TNM stage I tumor tissue; c, TNM stage II tumor tissue; d, TNM stage III tumor tissue; e, TNM stage IV tumor tissue. **(B)** Immune-histochemical scores of MICA/B in HCC tissues of different stages. Data were presented as means ± S.D., *P < 0.05, **P < 0.01.

MICA/B expression pattern was evaluated by immunohistochemistry in a retrospective cohort of HCC patients after tumor resection. Among the 96 patients (Additional file 1: Table S1), 75 patients (78%) showed positive MICA/B expression in the cytoplasm and membrane (Figure 1A). The other 21 patient samples showed no detectable MICA/B expression. Additionally, MICA/B was not detected in the surrounding non-cancerous tissues and normal hepatocellular tissues (Figure 1A). We also evaluated the immunohistochemical score of MICA/B expression. It was found that the expression level of MICA/B was significantly higher in the hepatoma cells at early stages (stages T1 and T2) compared to that of the hepatoma cells at advanced tumor stages (stages T3 and T4) (Figure 1B). These results suggested that MICA/B expression was significantly and negatively associated with TNM stage in hepatocellular carcinoma.

### Expression level of MICA/B was negatively associated with the clinical outcome of HCC

**Figure 2:**
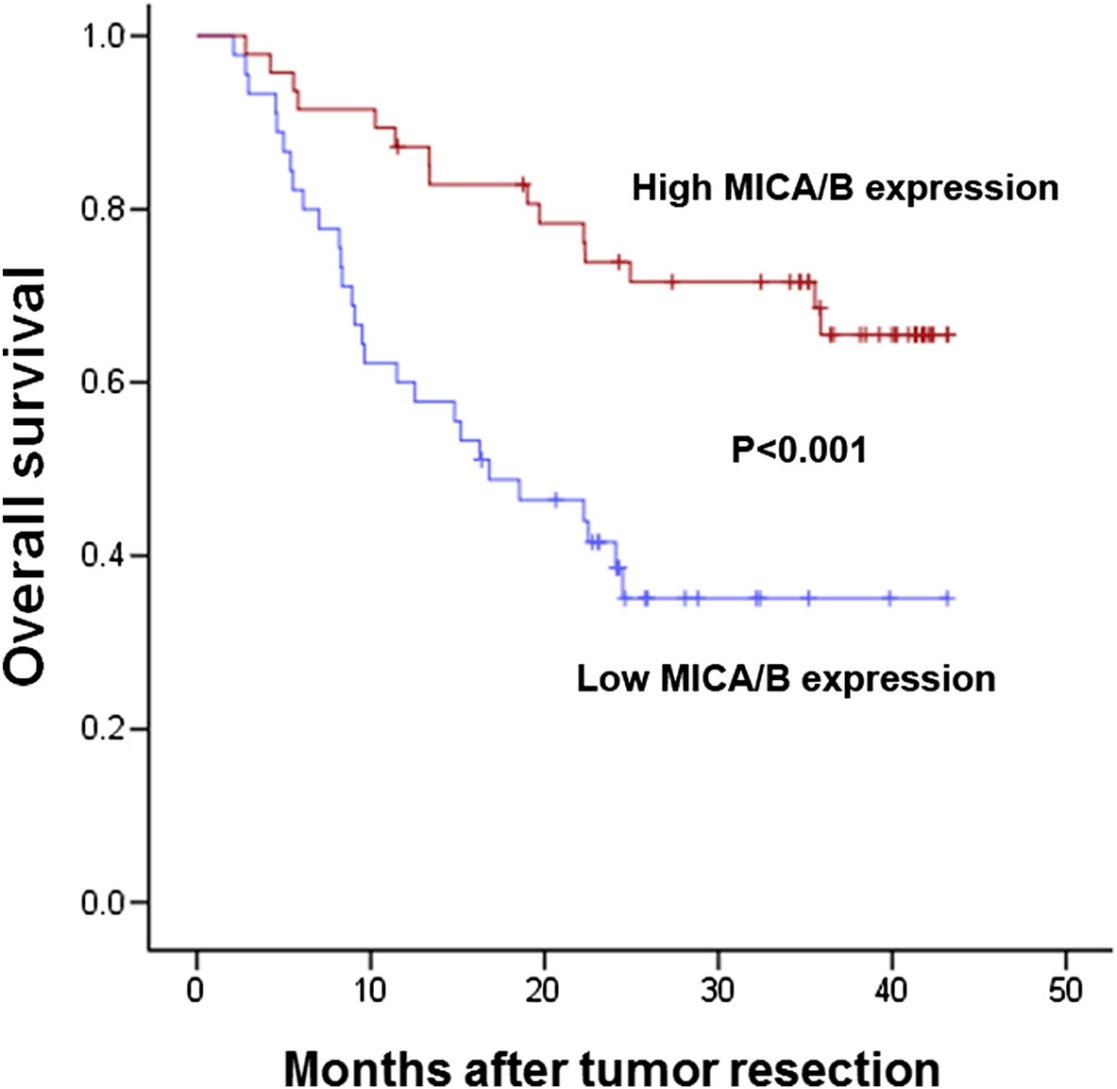
**Kaplan-Meier survival curves of HCC patients with different MICA/B expression levels.** Patients with high expression level of MICA/B had a better overall survival than those with low expression level of MICA/B (log-rank test, P < 0.001).

To determine the prognostic value of MICA/B expression in hepatocellular carcinoma, the HCC patients were divided into two groups by the MICA/B expression score. Kaplan-Meier survival analysis was done to explore the relationship between MICA/B expression level and patient survival. These results showed that patients with high expression level of MICA/B had a longer overall survival than those with low level of MICA/B ( log-rank test, *P* < 0.001) (Figure 2).

### The expression of GRP78 was negatively correlated with MICA/B expression level

**Figure 3:**
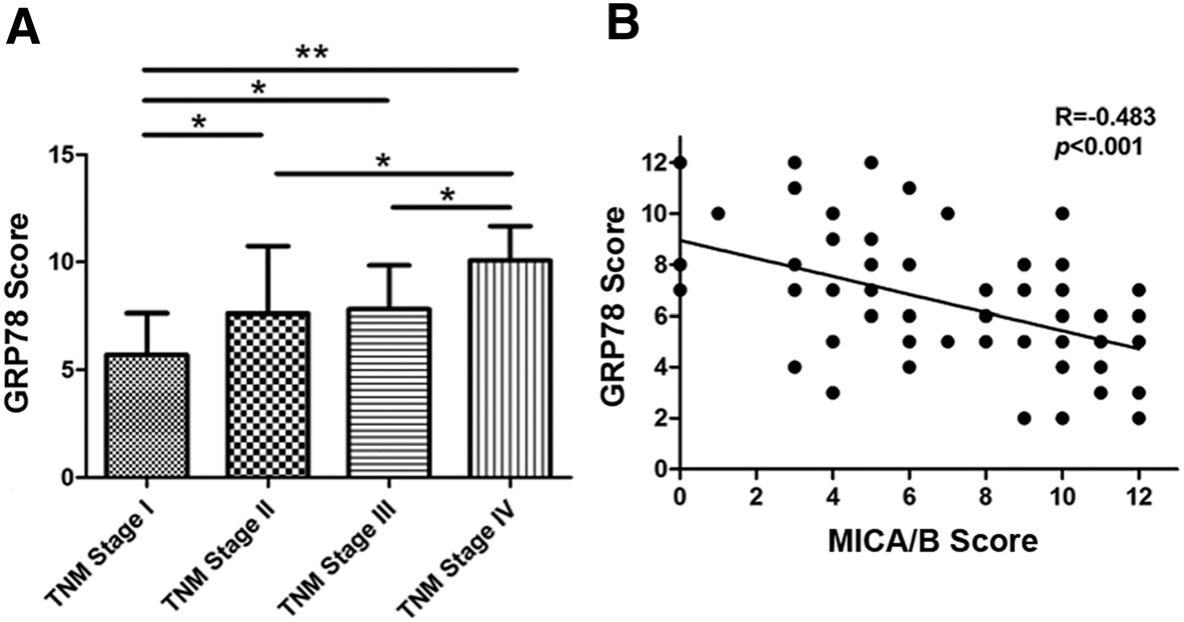
**Correlation of GRP78 and MICA/B expression levels in HCC patients. (A)** Immunochemical staining scores of GRP78 expression in human hepatoma tissues. Data were presented as means ± S.D., *P < 0.05, **P < 0.01. **(B)** Correlation between GRP78 scores and MICA/B scores in hepatoma tissues. *P* < 0.001.

HCC progression is often accompanied by hypoxia, decreased glucose supply and increased UPR in the tumor microenvironment. As expected, the expression level of GRP78, a molecular chaperone involved in the UPR, was increased in the hepatoma tissues and significantly related with tumor TNM stage (Figure 3A). In contrast, the GRP78 level was negatively correlated with MICA/B expression level (coefficient, -0.483; *P* < 0.001) (Figure 3B).

### UPR downregulated MICA/B expression in HCC

**Figure 4:**
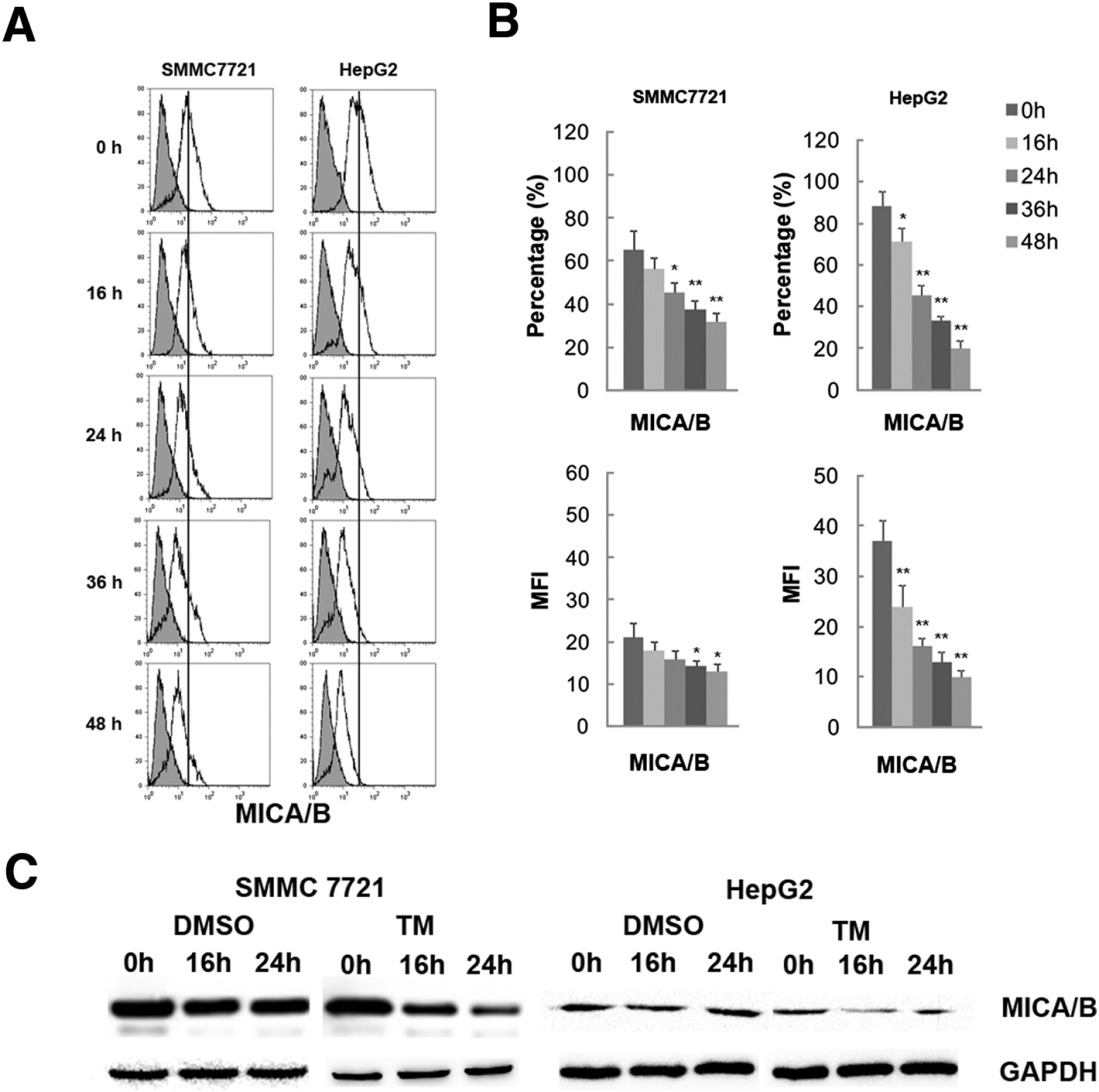
**Decreased expression level of MICA/B on hepatoma cells following tunicamycin(TM) treatment. (A)** Surface expression level of MICA/B on SMMC7721 and HepG2 cells was analyzed using flow cytometry. The grey histograms represent the isotype control staining. **(B)** Upper panel: data on the y-axes represent the mean percentage positive staining. Lower panel: data on the y-axes represent the mean florescence intensity. Data were presented as means ± S.D., *P < 0.05, **P < 0.01. **(C)** Total protein expression level of MICA/B in SMMC7721 and HepG2 cells treated with DMSO or TM for 16 or 24 h were analyzed using a western blot assay. GAPDH was detected as a loading control. The data shown are representative of three individual experiments.

To test the potential effect of HCC UPR on MICA/B expression, SMMC7721 and HepG2 hepatoma cell lines were treated with tunicamycin (TM, a naturally occurring antibiotic that induces ER stress by inhibiting the first step in the biosynthesis of N-linked oligosaccharides in cells ) for indicated times. Membrane-bound MICA/B was consistently expressed at high levels in SMMC7721 and HepG2 cells. TM treatment resulted in a gradual decrease of their expression beginning at 16 hour post-treatment. A significant reduction in both cell lines was observed at 36 and 48 hour as measured by both the percentage of stained cells and by MFI (Figure 4A, and B). Protein expression levels of MICA/B detected by western blot showed a similar pattern to that of the membrane-bound molecules detected by flow cytometry (Figure 4C). These results revealed that the UPR down-regulated total and membrane protein expression of MICA/B.

### Decreased expression of MICA/B occurred via post-transcriptional regulation

**Figure 5:**
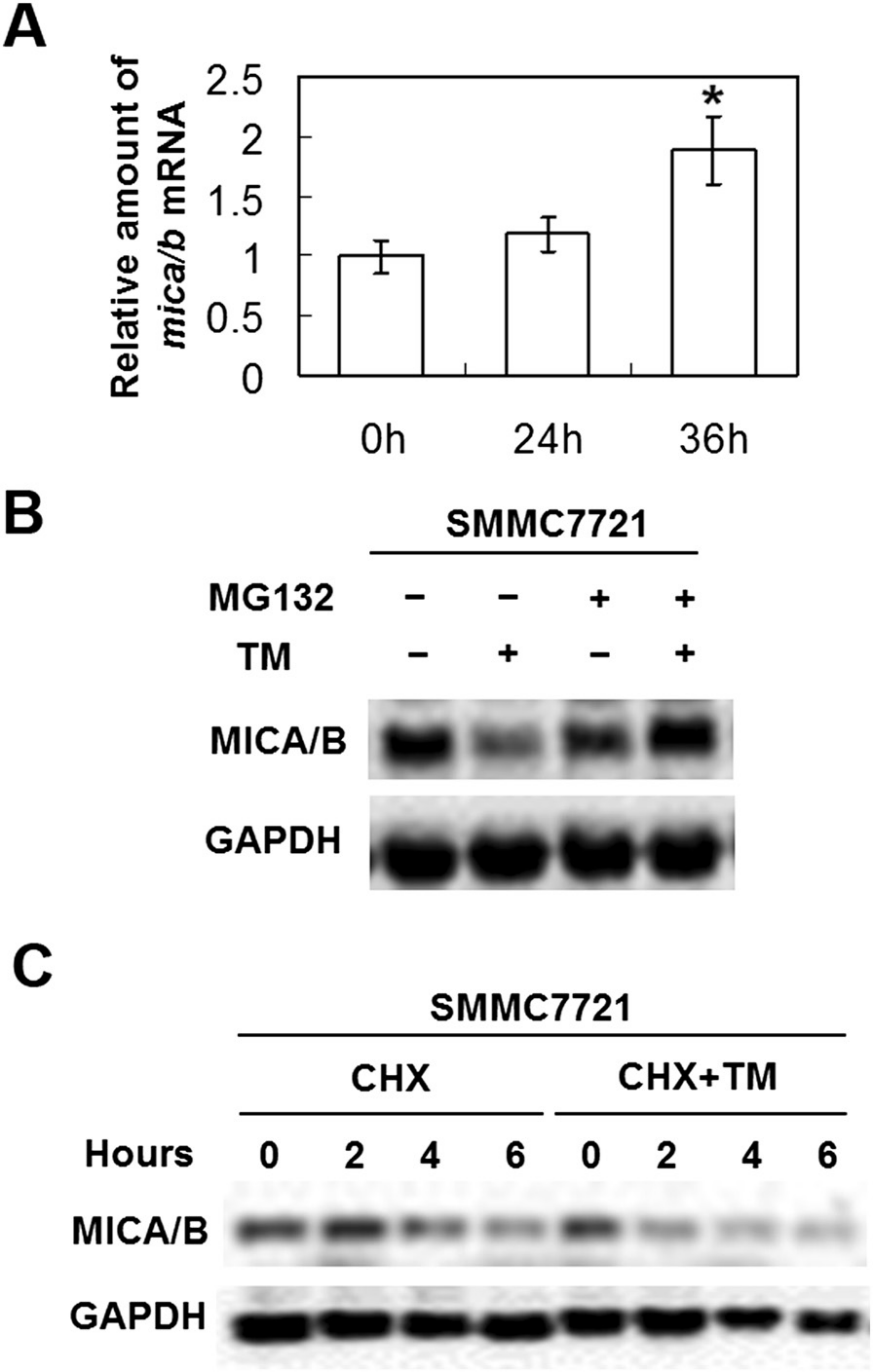
**Protein degradation was a mechanism for decreased MICA/B protein expression during UPR activation. (A)** Increased expression level of MICA/B mRNA in SMMC7721 cells after the treatment with TM for 24 h and 36 h. Bars represented the relative quantities of MICA/B mRNA in SMMC7721 cells (mean and S.D.). *P < 0.05. **(B)** The proteasome inhibitor MG132 reversed the downregulation of MICA in hepatoma cells during UPR. SMMC7721 cells with or without pretreatment with the proteasome inhibitor MG132 (10 mM) for 1 h were treated with TM (3 mM) for 24 h. Total protein from whole-cell lysates was then subjected to western blot analysis for MICA/B and GAPDH (as a loading control). The data shown are representative of three individual experiments. **(C)** UPR accelerated the turnover rate of MICA/B in hepatoma cells. SMMC7721 cells were treated with the protein synthesis inhibitor cycloheximide (CHX; 10 mg/ml) with or without the addition of TM (3 mM) for the indicated periods. Total protein from whole-cell lysates was then subjected to western blot analysis for MICA/B and GAPDH (as a loading control). The data shown are representative of three individual experiments.

When the mRNA level of MICA/B was analyzed, TM treatment unexpectedly caused a gradual increase of MICA/B mRNA level with a significant increase at 36 h in SMMC7721 cells (Figure 5A). These results suggested that their decreased protein expression did not result from lowered transcript level, and might have occurred via post-transcriptional regulation. We investigated whether the protein degradation was a mechanism for decreased MICA/B protein expression during UPR activation. It was found that the proteasome inhibitor MG132 inhibited the downregulation of MICA/B protein in SMMC7721 cells induced by TM (Figure 5B), indicating that the decreased expression of MICA/B in HCC during UPR was associated with proteasomal degradation. In support of this possibility, protein half-life analysis in the presence of the protein synthesis inhibitor cycloheximide (CHX) showed that the MICA/B turnover rate was more rapid in SMMC7721 cells treated with TM as compared to cells exposed to DMSO (Figure 5C).

### MICA/B downregulation by UPR is linked to reduced NK cell–mediated cytotoxicity

**Figure 6:**
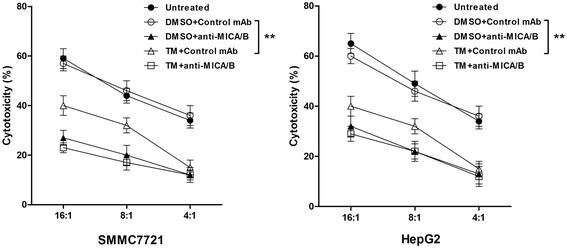
**Reduced cytotoxic activity of NK cells against hepatoma cells treated with TM for 24 h.** The susceptibility of SMMC7721 cells (left) and HepG2 cells (right) to NK cell cytolysis was determined using the LDH cytotoxicity assay. The NK cells were incubated with SMMC7721 or HepG2 cells at the indicated effecter/target ratios. Data were presented as means ± S.D., **P < 0.01.

Because changes in the expression of NKG2D ligands (such as MICA/B) on tumor cells can modify the recognition and activation of NK cells via NKG2D, while the previous result showed that TM reduced MICA/B levels, we next tested whether treatment of HCC cells with TM could also lead to decreased activation and NK cell-mediated killing. The susceptibility of SMMC7721 cells and HepG2 cells to NK cell cytolysis was determined using the LDH cytotoxicity assay. We observed that TM treatment reduced NK cytoxicity against SMMC7721 and HepG2 cells. To confirm this decrease of cytotoxicity was dependent on NKG2D/MICA/B interaction, we used a blocking anti–MICA/B mAb to mask the cell-surface MICA/B. This treatment significantly inhibited the cytolytic activity of NK cells against SMMC7721 and HepG2 cells in comparison with that of control mAb, but the differences of cytolytic activity between TM and DMSO groups could be reduced (Figure 6). These results suggested that the down-regulation of MICA/B expression level by the activated UPR weakened the killing activities of NK cells against HCC.

## Discussion

Major histocompatibility complex class I-related chain A and B (MICA/B) are stress-inducible ligands that bind to the immunoreceptor NKG2D and play an important role in tumor immunity [16]. In humans, MICA/B is rarely expressed in healthy cells, but a broad expression in carcinoma cells, such as colorectal [17,18], breast [19], pancreatic cancers [20] and hepatocellular carcinoma [21]. Previous studies showed that the serum level of soluble MICA (sMICA) is an independent prognostic factor for advanced hepatocellular carcinoma [22], and the interaction of NKG2D and corresponding ligands MICA/B may play an important role in immune-surveillance against cholangiocarcinoma and hepatocellular carcinoma [23,24]. However, the correlation between hepatocellular carcinoma progression and membrane-bound MICA/B expression has not been well explored.

In line with previous studies, we confirmed that the expression of MICA/B could be detected in HCC cells and negatively associated with HCC TNM stage. We also provided evidence that low expression levels of MICA/B in human hepatoma tissues were significantly associated with a poorer outcome in patients with HCC. Our results showed the patients with low MICA/B expression are usually at a significantly higher risk of tumor progression and shorter overall survival.

*Previous studies documented that the expression of NKG2D ligands are up-regulated through a variety of stimuli that have been collectively termed “cellular stress”, such as cellular transformation, viral infection, and/or DNA damage* [25,26]*, and the upregulation of MICA/B in distressed cells may alert the immune system that the cells are undergoing pathological changes and enhance the innate immune function. However there has been no report of whether the UPR in HCC could regulate the expression levels of the ligands for NK cell activating receptors and affect the sensitivity to NK cell cytolysis. It is well known that the rapid proliferation of HCC coupled with poor blood supply usually leads to hypoxia, decreased glucose, nutrition supply and UPR in tumor tissues during HCC progression* [11]*. Acting as a cytoprotective response against malignant cells, the UPR reduces the accumulation of unfolded cellular proteins and restores normal ER function. Our study represents the first demonstration that activated unfolded protein response (UPR) could down-regulate the protein expression level of MICA/B in HCC cells. Besides, another important finding of the present study is that GRP78, a marker of UPR, was significantly related with tumor TNM stage and negatively correlated with MICA/B expression level in HCC tissues. This finding suggested that activation of the UPR was negatively correlated with MICA/B expression in HCC tissues and consistent with the results from HCC cell lines.*

Although the treatment of different hepatoma cell lines with the ER stress inducer tunicamycin reduced the protein levels of MICA/B on hepatoma cells, this is not accompanied by a reduction of MICA/B mRNA during the UPR. We conjectured that the inhibition of protein expression was likely mediated by post-transcriptional mechanisms. Further experiments confirmed this conjecture: the reduction of MICA/B expression in hepatoma cells during the UPR was at least partly due to proteasomal degradation. However, the regulation mechanisms responsible for the upregulation of MICA/B transcription require further investigation.

To further clarify the function of MICA/B in hepatoma cells during UPR, we assessed if reduced expression level of MICA/B on hepatoma cells undergoing UPR had any effects on NK cells cytotoxicity against hepatoma cells. The result showed that TM could attenuate the activity of NK cells against the tumor, via mechanisms that can involve a parallel action on NK cells and malignant cells, decreasing the expression of activating ligand that promote recognition and reducing the cytolytic activity of the effect cells.

It should be noted that loss of the predominant membrane-bound MICA/B is associated with progression to invasive tumor or to progressively higher grades [27]. On the other hand, high MICA/B expression is inversely correlated with survival in patients with pancreatic carcinoma [28]. These reports suggest a fundamental difference in the involvement of MICA/B -mediated immunity among various types of tumors.

In conclusion, we provide novel information about MICA/B in HCCs. Our results showed the HCC patients with low MICA/B expression are usually at a significantly higher risk of shorter overall survival. Furthermore, UPR could downregulate MICA/B expression on hepatoma cells, and thereby rendered them less susceptible to NK cytolysis. This suggests that reducing UPR may have an impact on increasing MICA/B expression and enhancing NK cytotoxicity and thus may be an useful strategy in treatment of HCC.

## Additional file

Additional file 1: Table S1. Clinical pathological characteristics of 96 HCC cases.

## Abbreviations

UPR: Unfolded protein response
MICA/B: MHC class I–related chain A/B
HCC: Human hepatocellular carcinoma
GRP78: Glucose-regulated protein of 78 kDa
TM: Tunicamycin
