# Supplementary material for: MICA/B expression is inhibited by unfolded protein response and associated with poor prognosis in human hepatocellular carcinoma
Source: J Exp Clin Cancer Res. 2014 Sep 18;33(1):76. doi: 10.1186/s13046-014-0076-7 (PMC4174668; doi:10.1186/s13046-014-0076-7)
Supplement: Additional file 1: Table S1. — Clinical pathological characteristics of 96 HCC cases. [file 13046_2014_76_MOESM1_ESM.docx]

Table S1. Clinical pathological characteristics of 96 HCC cases

| **Variables** | **Case number (%)** |
| --- | --- |
| **Age (years)** |  |
| <=52 | 50 (52.1) |
| >52 | 46 (47.9) |
| **Gender** |  |
| Female | 18 (18.8) |
| Male | 78 (81.2) |
| **Chronic HBV infection** |  |
| Yes | 95 (99.0) |
| No | 1 (1.0) |
| **AFP (μg/ml)** |  |
| <200 | 52 (54.2) |
| >=200 | 44 (45.8) |
| **PVTT** |  |
| No | 73 (76.0) |
| Yes | 23 (24.0) |
| **TNM stage** |  |
| I | 40 (41.7) |
| II | 23 (24.0) |
| III | 28 (29.1) |
| IV | 5 (5.2) |
| **Differentiation** |  |
| Well | 5 (5.2) |
| Moderate | 36 (37.5) |
| Poor | 55 (57.3) |
| **Survival status** |  |
| Alive | 51 (53.1) |
| Dead | 45 (46.9) |

HBV: hepatitis B virus; AFP: alpha fetal protein; PVTT: portal vein tumor thrombosis.
